# Exploring patient and public involvement (PPI) and co-production approaches in mental health research: learning from the PARTNERS2 research programme

**DOI:** 10.1186/s40900-020-00224-3

**Published:** 2020-09-21

**Authors:** The PARTNERS2 writing collective, Dawn Allen, Lindsey Cree, Paul Dawson, Shaimma El Naggar, Bliss Gibbons, John Gibson, Laura Gill, Ruth Gwernan-Jones, Charley Hobson-Merrett, Beverly Jones, Hameed Khan, Catherine McCabe, Mary Mancini, Dougie McLellan, Mary Nettle, Vanessa Pinfold, Tim Rawcliffe, Angela Sanders, Ruth Sayers, Deb Smith, Diane Wright

**Affiliations:** 1 McPin Foundation, London, UK; 2 Lancashire Care NHS Foundation Trust, Lancashire, UK; 3 University of Birmingham, Birmingham, UK; 4 University of Plymouth, Plymouth, UK; 5 University of Exeter, Exeter, UK

**Keywords:** Experiential expertise, Collaborative methodologies, Reflective accounts, Service user researcher, Cooperative inquiry

## Abstract

**Background:**

Patient and Public Involvement (PPI) in research is a growing field of work, incorporating experiential knowledge within research processes. Co-production is a more recent PPI approach that emphasises the importance of power-sharing to promote inclusive research practices, valuing and respecting knowledge from different sources, and relationship building. Applying co-production principles in research trials can be difficult, and there are few detailed worked examples or toolkits. This paper explores the successes and challenges encountered by one research team.

**Methods:**

Our paper is written by a team of 21 people working on PARTNERS2, led by a smaller co-ordinating group. Using a co-operative style inquiry, the authors have reflected on and written about their experiences; analysis of the resulting 15 accounts provided examples of how PPI and co-production were delivered in practice.

**Results:**

We reveal varied and complicated experiences as we developed our collaborative approach across the entire research programme. Four main themes emerge from reflective accounts which describe aspects of this process: (1) recognising the importance of ‘emotional work’; (2) developing safe spaces to create and share knowledge; (3) some challenges of using our personal identities in research work; and (4) acknowledging power-sharing within the research hierarchy. We also found continual relationship building, how different forms of expertise were valued, and stigma were central to shaping what work was possible together. Other important practices were transparency, particularly over decision making, and clear communication.

**Conclusions:**

Our work provides one example of the ‘messy’ nature of collaborative research in practice. The learning we surface was contextual, generated within a large-scale research programme, but applicable to other studies. We found for success there needs to be an acknowledgement of the importance of emotional work, creating safe spaces to co-produce, transparency in decision making and reflection on the difficulties of using personal identities in research work including for service user researchers. These elements are more important than existing guidelines suggest. Implementation of actions to support emotional work, will require changes within individual teams as well as institutions. Introducing reflective practice in teams may be helpful in identifying further improvements to inclusive research practice.

## Plain English summary

PARTNERS2 is a study funded to develop new ways of supporting people with schizophrenia, bipolar or other psychoses in primary care. This paper describes our experiences of working together as service users, carers, and researchers. The aim was to explore our approach to integrating expertise from lived experience and offer learning that may apply elsewhere. We produced 15 written accounts, 11 by individual authors and 4 by writing teams, describing examples of work we did together. Analysing the accounts, we identified four key themes that explore our work in practice: i) recognising and dealing with emotions in the workplace; ii) the importance of developing safe spaces; iii) some challenges to using our personal identities in research work; and iv) aspects of sharing power within university-based research systems. We also found that relationship building, how different forms of expertise were valued, and mental health stigma were also central to shaping what work was possible together. We highlight how difficult collaborative research can be in practice, with particular challenges for service user researchers working on research trials. Other important practices were transparency, particularly over decision making, and clear communication. Published principles for both ‘patient and public involvement’ and ‘co-production’ were observed. However, we emphasise the importance of supporting emotional work, for both advisors and researchers, an element that is more important than guidelines suggest. We found introducing reflective practice was helpful in identifying changes needed to improve involvement and co-production work, creating more inclusive research practices.

## Background

Health research funders increasingly require evidence that potential beneficiaries have meaningfully contributed to the development of studies [1, 2], including mental health studies [3, 4]. In the UK, this is mainly known as Patient and Public Involvement (PPI) [5]. Descriptors such as lay advisors and public contributors are commonplace but the roles undertaken in practice vary and require different skills [6]. There is less agreement on how survivor and service user researchers are positioned in research frameworks as distinct from PPI or part of the involvement narrative [7].

The UK has new PPI research standards: inclusive opportunities; working together; support and learning; communications; impact and governance [8]. Yet there remains a lack of clarity around what PPI is, and how it operates in practice [9–11]. Although its value base is defined [12], its internal coherence as an entity has been challenged [13].

Co-production has been promoted more recently as an approach to public involvement in research [14], imported from service development [15, 16]. It provides a template of values and principles for working towards greater equality within research teams [17], co-producing knowledge [18]. Transitioning to research practice is not straightforward [19]. Co-production demands that researchers work differently both scientifically and practically, relinquishing and sharing established power bases [20]. The literature on co-production is growing [21], however, few studies have explored how co-production is achieved in practice [22–24]. This paper aims to contribute to emerging literature on integrating experiential expertise using PPI and co-production by reflecting upon the experiences of one research team, offering learning that may apply elsewhere.

### The PARTNERS2 programme

We work on a multi-site complex research programme including a clinical trial, funded by the National Institute for Health Research (NIHR) 2014–2021. The programme’s focus is collaborative care for people with a primary diagnosis of schizophrenia, bipolar or other psychoses [25]. Some authors have an expert from experience background, some a researcher background, and some both. PARTNERS2 actively works to fill positions across the research team by combining academic, clinical, and experiential expertise. Our involvement plan includes roles for mental health service users as advisors, researchers and in leadership positions.

Delivery of the involvement plan, using both PPI and co-production approaches, is supported by the McPin Foundation, a charity employing two staff members in the programme team and running LEAPs (Lived Experience Advisory Panels). The plan is guided by the National Survivor User Network (NSUN) involvement standards (4PI): principles, purpose, presence, process and impact [26]. Collaboration is inherent in the PARTNERS2 intervention, and we aim to reflect this in our involvement work and research delivery. Over time, many people working on PARTNERS2 but not formally part of the ‘PPI team’ came to also disclose their mental health experiences providing more experiential expertise than initially planned.

## Methods

**Fig. 1:**
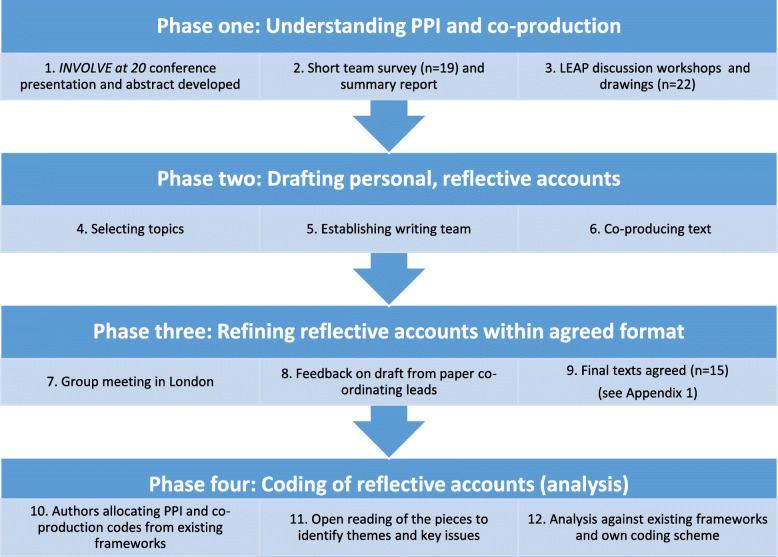
Overview of approach: writing about PPI and co-production

This paper utilises co-operative inquiry to gather reflective accounts from team members [27]. The approach chosen is inclusive and based upon principles of collaboration, thus fitting the project’s ethos. We comprise 11 members of our three LEAPs, eight researchers (some of whom were service user researchers), the PARTNERS2 PPI Lead and PPI Co-ordinator/peer researcher. To ensure the task was manageable, 10 co-applicants and senior staff did not contribute. Co-operative inquiry requires reaching a consensus and exploring divergent viewpoints. This can be challenging, and during the preparation of this paper our learning changed. We undertook 12 stages of reflection and self-inquiry (Fig. 1).

### Phase one: planning

First, we focused on involvement experiences and the concepts of PPI and co-production without providing definitions. We circulated a bespoke survey to LEAP members. The summary was used by team members (RS and JG) to run three one-hour workshops, one in each research site. We used creative methods [28] to draw personal images representing both PPI and co-production. These enabled discussions of the emotions that lie beneath concepts and practices. We shared the drawings with each other; indicating those that resonated most and feeding back by writing captions. We discussed a range of themes, issues and challenges that could form reflective writing topics. Authors chose their own topic and writing partners.

### Phases two and three: writing

We wrote reflective accounts, illustrating how we worked together. Drafts were completed prior to a planning meeting (stage 7), attended by 14 authors (7 LEAP members, 7 researchers). The drawings (stage 2) were displayed to aid discussion while we worked in small groups to suggest elaboration and refinements to the written accounts. We also agreed a preliminary structure for the paper.

Fifteen reflective writing accounts were completed, 11 from individual authors and four from co-author teams (see Appendix 1). Authors coded their accounts using published PPI and co-production guidance [8, 14]. This deductive approach enabled us to compare how well our own experiences tallied with the principles of established frameworks.

### Phase four: analysis

**Fig. 2:**
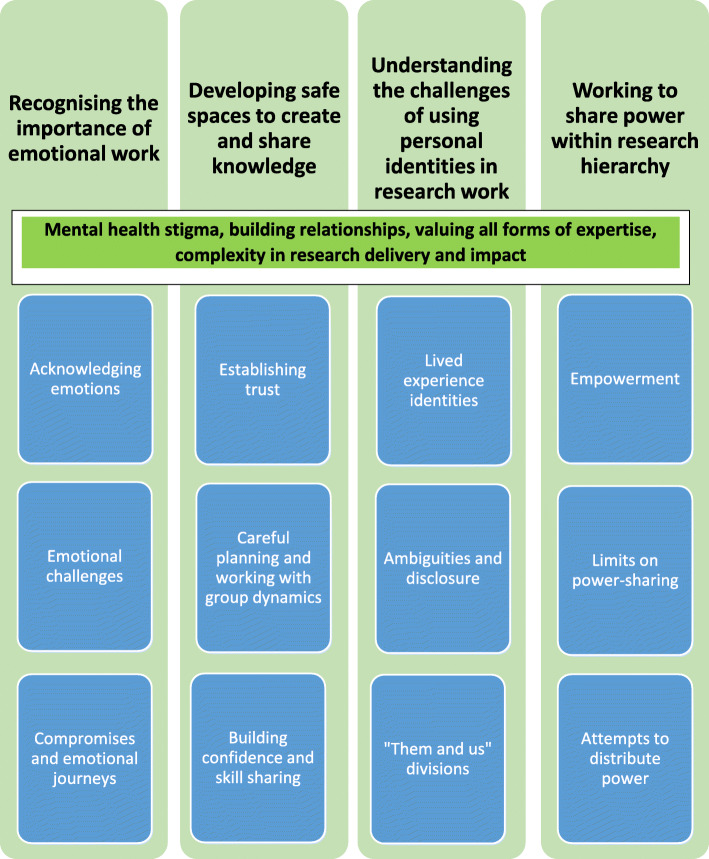
Reflective writing accounts: co-author coding scheme

Analysis was based on written accounts. We adopted a pragmatic inductive approach [29] aiming to explore our experiences and understanding of PPI in PARTNERS2. The co-author group meeting (stage 7) (RS, VP, CM) generated ideas for key messages which consisted of five overarching themes. Sub-themes emerged (see Fig. 2), confirmed by further reading and discussions.

## Results

Our experiences are presented through four themes and sub-themes (see Fig. 2). Although distinct these categories overlap; relationship building, the impact of stigma and how different expertise was valued were all particularly consistently addressed in accounts. The context of our work was a multi-site clinical trial that was experienced as ‘complex’ including being granted a funding extension in 2019.

### Recognising the importance of emotional work

#### Acknowledging emotions

Understanding why and how relationships are central to involvement work required awareness of, and attending to, emotions with which people arrive, and those that are stimulated by the work itself, regardless of a person’s project role or title.

*I am not an academic. I am a mother wading my way through the mental health system, so it’s an emotional journey too. (Account B, LEAP member).*

Not everyone wanted to share emotions openly, while others welcomed the opportunity to do so. Understanding people’s preferences was part of team development.

*I learnt to develop respect for the differences that existed and for some that might mean more disclosure than for others. (Account D, LEAP member).*

#### Emotional challenges

Using personal experiences in PARTNERS2 challenged emotions in different and unexpected ways. One researcher found it surfaced complicated feelings about their past.

*The role and identity of being a ‘service user researcher’ on PARTNERS2, has at times created an internal tension for me, particularly with the transition of the project to a site where I’d previously accessed secondary care services. Once I would have been the one waiting in the reception area of the Community Mental Health Team; I have felt at times that I am now ‘on the other side’. (Account N, SURA).*

Our collective emotional safety relied on people accepting different approaches to using lived experiences and recognising the varied professional skills, that both LEAP members and research staff, brought to the project.

*I remember being frustrated with one-member sharing details about their family member …*. *I felt very judgemental and resentful that they were taking up time sharing; I did not think it was what we were there for … (Account D, LEAP member).*

#### Compromises and emotional journeys

Work as service users and carers can never be hazard-free and involves compromises.

*We were trying to decide on which outcome questionnaires participants would be completing. I felt very conflicted. One of the favoured questionnaires asked about work and claiming benefits. I could see from a researcher point of view why this choice made sense. But I felt I was betraying service users because recording outcomes about benefits and work-related issues can cause stress and raise fears that people might lose benefits. (Account K, LEAP member).*

Where perspectives from lived and academic experience conflicted, this could be emotionally draining. Research staff also found LEAP meetings emotionally challenging.

*[LEAP members] emphasised the need for substantial changes …*. *I found the comments difficult to hear. I think because I had worked hard on the resources, following the empathetic stance I developed. It was humbling to see the benefit of the changes I had been blind to before the LEAP suggested them. (Account I – Research Fellow).*

The journey also included periods of absence and returns to work for some. Learning from mistakes was key to our project.

*For me personally, [it has been] a roller-coaster ride, with handrails that, at first, were not screwed in properly. But, from every bump and crash, we learned how to find our balance and not risk tipping the whole thing over. Learning to be flexible, but mindfully. (Account G, PPI Co-ordinator and Peer Researcher).*

### Developing safe spaces to create and share knowledge

#### Establishing trust

To become fully involved and contribute experiential perspectives to research team members needed to feel safe (psychologically and physically) which required open and trusting environments within PARTNERS2.

*As LEAP members we were viewed as having skills and a unique perspective. Engaging with each other, researchers, and administrative staff, produced new insights, trust-building and created a positive working environment. (Account H, LEAP members).*

Initially most contact was face-to-face meetings; LEAP applicants met local research staff and each other. For service user researchers, the initial phase included establishing relationships with colleagues in three different employing organisations: an NHS Trust, a University department, and a research charity.

#### Careful planning and working with group dynamics

Practical arrangements and settings for meetings were important, affecting relationship formation and the meeting atmosphere. This affected how research staff felt, and whether they shared relevant personal experiences or not.

*I assumed a role of academic researcher and felt both connection and disconnection to other people around me [because of my own mental health experiences]. There was so much discussion and work around PPI in the project that this position of being “Inside Out” felt very strange. (Account O, Research Assistant).*

LEAP groups developed different cultures, arising from relationships between individual members, group dynamics and the venue. One LEAP member, moved groups after relocating and stated:

*The first LEAP felt like a family and was based in a community venue, almost like walking into a neighbour’s house. It felt safe. The second LEAP feels much more formal. Meetings are held on secure university premises. We work together as colleagues, rather than family. (Account E, LEAP member).*

We also sought opportunities to bring people together for key decision-making sessions, such as selection of trial outcome measures: co-applicants, research staff, and LEAP members. To encourage reciprocal discussions, we carefully planned meetings.

*[we tried to involve everyone by] altering seating arrangements in team meetings to mix people up; exercises to equalise power in meetings, sharing personal interests and skills; collecting feedback to revise processes. (Account A, PPI Lead).*

#### Building confidence and skill sharing

Establishing stable, inclusive, and friendly groups helped equip LEAP members for difficult aspects of their roles. Challenges included being unfamiliar with the research processes, including concepts and language (terminology, acronyms, and jargon).

*At times I felt hugely out of my depth. (Account K, LEAP member).*

LEAP members were conscious that their experiences added valuable perspectives to research. Lack of confidence worried people, but over time capacity to engage grew. Several people brought other transferable skills.

*We also learnt from each other and acquired skills to use in other roles, including trustee of a local drop-in centre. (Account H, LEAP members).*

### Understanding the challenges of using personal identities in research work

#### Lived experience identities

For both LEAP members and researchers, drawing upon expertise from experience to contribute to research explicitly requires the individual to be open about an identity they hold. We brought many additional identities to our research work, some linked directly with mental distress, others not. There is, however, a danger of not allowing or facilitating all these identities, which can leave people feeling only partially engaged or useful.

*I joined a PARTNERS2 LEAP as someone who has used mental health services, but I also brought other identities, some more visible than others: carer for my mum, ethnic minority background, gay man, Muslim …*. *Research will benefit from including all those perspectives, but only if I feel safe, and can choose whether, and how, to reveal them. (Account E, LEAP member).*

#### Ambiguities and disclosure

Researcher job descriptions did not always correspond with self-perceptions of identity, and placed limits on their overall contribution to PARTNERS2. Some lived experience expertise within the research team remained undisclosed, concealed behind a job title.

*Being labelled as a “normal” researcher negated my own insights from actually having mental health experiences - simply because it wasn’t in my job title or expected of me. This made me feel uneasy in the sense of being labelled as an outsider in our LEAP, by people in the group stereotypically labelled as outsiders themselves …*. *None of this was ever discussed openly. (Account O, Research Assistant).*

Using experiential expertise in a university department or an NHS facility workplace involved a risk for researchers, of being seen to occupy a lower position because of a mental health diagnosis. Managing this tension could be stressful.

*Being a ‘service user researcher’ has often seemed to work in many different and contrary ways within PARTNERS2. Sometimes as an asset, one example being a resource to draw upon in conversations with potential participants, including participating GP practices. At other times there has been a sense of appearing somehow of less ‘status’ within the academic hierarchy. (Account N, SURA).*

Such ambiguities had not been anticipated and could lead to strained relationships across the team, as well as internal struggles for individuals.

#### “Them and us” divisions

We became increasingly aware of disadvantages through labelling researchers as either part of the PPI team or not, unintentionally developing a ‘them and us’ division. Tasks became unnecessarily compartmentalised as either ‘academic’ or ‘personal experience’ work. Roles and identities overlapped.

*I draw on my personal experiences to contribute to many aspects of the study, as well as carrying out the tasks of an academic researcher. (Account F, LEAP member / SURA).*

The research team response to the service user researcher job title and associated identity varied. In one site researchers felt that having ‘Service User’ added to their ‘Research Assistant’ job title created unhelpful differences.

*There was a sense that the title of ‘service user researcher’ was too narrow. It could be detrimental to a researcher’s career, and lead to ignoring other qualifications and experience. (Account M, three Research Assistants).*

Thus, a strength of teamwork was the acceptance of ‘messiness’ in PARTNERS2 involvement work. This enabled deeper thinking: binary approaches to identity were challenged allowing team members to reflect on assumptions they made about each other.

*Although these sessions weren’t always easy - I felt judged as an academic for not being ‘one of them’ (Account I – Research Fellow).*

Positively, staff working openly with their own lived experience helped address gaps between different cultures, assumptions, knowledge, skills, and motivations that operated within the wider team, as experiential and professional expertise were exchanged.

*I feel it’s absolutely crucial we have service user researchers on board directly. Roles that form a bridge between service users/carers and academics. They understand where we are coming from. It could be said they wear two hats, bringing our motivational differences together … I think the service user researcher role reduces the gap between us and them in important ways. (Account B, LEAP member).*

### Working to share power within research hierarchy

#### Empowerment

Power differentials and hierarchy were recognised as barriers to achieving collaborative work in practice. This was not seen as insurmountable.

*Where possible, projects should strive towards mutual agreement between academic experts and those with ‘expertise from experience’. This reduces the perception of the superiority of academic and clinical knowledge over experiential knowledge. (Account L, Two LEAP members and a Research Assistant).*

Joining a research team was one way of making positive use of experiences through being heard and believed, feeling useful and wielding influence.

*Work within PARTNERS2 has enabled me to add value to my difficult health experiences and positively redefine ‘mental illness’, overcoming the detachment from a working society. (Account C, LEAP member).*

However, more could have been done to continually assess how to integrate different expertise across the team.

*It was empowering, but we could bridge the gap even further between academics and people with personal experience of the topic being researched. We saw few senior staff at the LEAP meetings, which was unfortunate as we could have learned a lot from each other. (Account H, LEAP members).*

Overall, this work valued and respected the expertise that each person brought to a project. Researchers gain expertise from LEAP members and vice versa.

*While I had understood the issues from reading literature, LEAP members’ stories about losing support that they depended on, and distress over losing relationships with trusted practitioners, brought the issue to sharp focus. (Account I, Research Fellow).*

#### Limits on power-sharing

Running a mental health research programme, brought into focus the challenges of co-producing work.

*I guess any project like PARTNERS2 is very complex and messy, and it certainly has been. (Account K, LEAP member).*

There are examples of positive involvement and shared decision-making but there are also examples where we struggled to co-produce.

*We seek collaborative and transparent decision-making, but can feel disconnected and disempowered by decisions taken. (Account F, LEAP member and SURA).*

Due to the inherent power imbalance within research, our involvement plan did not allow PPI to disrupt established research culture, limiting its impact.

*Individual ‘breakthroughs’ in reciprocity and relationship building can feel short-lived …*. *We have no shortage of ideas or goodwill but changing research cultures fundamentally remains a huge challenge. (Account A, PPI lead).*

However, actualities of pragmatic compromise in our work were acknowledged.

*[As a service user researcher,] I am much closer to day-to-day decision-making. In theory, I have more ‘power’ to shape and direct the project. However, the practicalities of my role have made me far more aware of the need for compromise and pragmatism. (Account F, LEAP member / SURA).*

Involving everyone in everything was not feasible. Agreeing a clear framework for decision-making and transparent rules and practices was helpful, but in our experience not a complete solution.

*LEAP members were included at all stages of the project, but as always the research process gave members the impression of being listened to but not having the final say. This was partly due to the logistics of quarterly meetings, but also a result of academic hierarchy. (Account L, Two LEAP members and a Research Assistant).*

#### Attempts to distribute power

Across the study, efforts were made to address power imbalances. For example, LEAP members chairing meetings. Successful outcomes from collaboration included, the development of the study website (content, design, video scripts), selecting the trial primary outcome and developing a core outcome set. The latter two both involved consensus workshops with iterative voting and extensive discussion. This power sharing involved acknowledging different experiential, ideological and epistemological positions within the team.

*[Some of the researchers and I] were working from different starting points in terms of expertise on methods, mental health and activism. I advocated for broader conceptualisation - from ‘illness’ towards ‘well-being’. (Account G, PPI Co-ordinator and Peer Researcher).*

Commitment to shared decision-making, at times caused confusion and necessitated extended communication. This operated at several different levels but aimed to be transparent. Over time, the limits of decision-making were recognised and appeared accepted. This may have been due to ongoing development of trust.

##### Assessing our progress

**Fig. 3:**
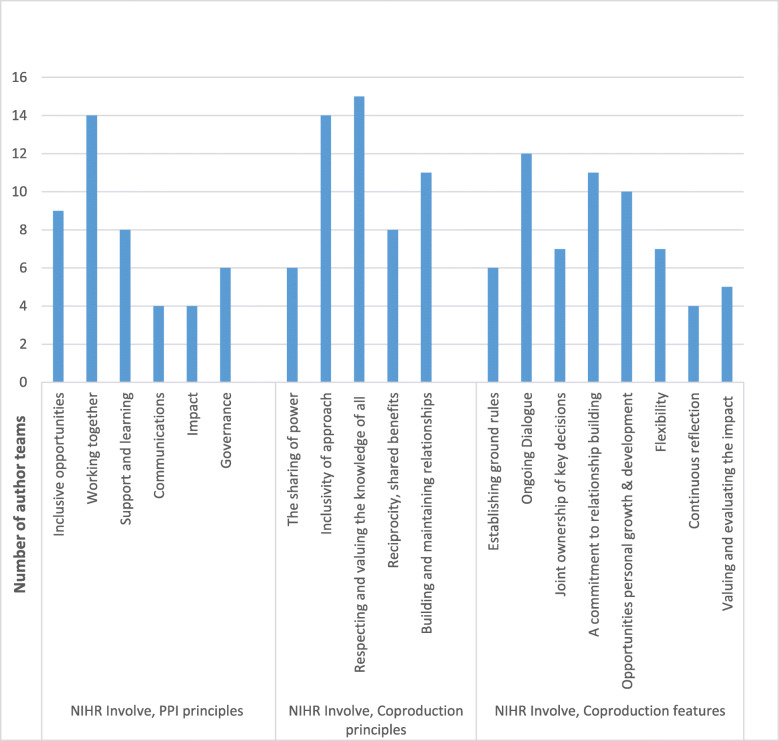
Author assessment of accounts against published frameworks for PPI [8] and co-production [14]

We compared our experiences against established PPI and co-production principles (see Fig. 3). Most of our accounts emphasised working together, inclusive approaches, respecting as well as valuing knowledge from all involved, open discussion, and relationship building. We wrote less about impact, communications, governance, and ground rules, although several accounts clearly focussed upon power-sharing.

## Discussion

PARTNERS2 had a substantial involvement plan to bring experts from experience into the research process. On considering recently published standards for PPI [8] and co-production [14] we can ‘tick the box’ that these are considered and some progress towards achieving them made. Below the surface is a complicated story of involving service users and carers in research, and their experiential expertise particularly in the service user researcher roles.

### Our learning: lived experience expertise

We focused on recruiting specific experiential expertise in the LEAPs and research team to improve the project in terms of research management and delivery. We achieved this goal, but the process raises questions about how best to do this in practice. The context for reflecting on experiential expertise, is understanding what expertise people bring and how it is applied to co-produce new knowledge. We did not do this enough in the first 3 years of the study. It is important that the term ‘people with lived experience’ is explored more fully to avoid ‘strategic essentialism’ [30] undermining the value of experiential knowledge by objectifying it in a single cohesive category. Diversity is important; we did not talk or reflect about race, gender, or sexuality openly to explore influence on involvement spaces or knowledge production. We must also be aware of the risk of treating lived experiences as a commodity [11]. In PARTNERS2 we have learnt planning agendas, re-arranging seating layouts in rooms, and appointing service user researchers is insufficient without constant communication and reflection on how best to utilise collective team expertise, including lived experience.

Emotional work emerged as an important theme. Teams must recognise, and provide support for, the emotional work involved in drawing upon expertise from experience in daily research work and advisory groups. The cross-cutting theme stigma does impact in academia; we saw it manifested in feelings of ‘lower status’ by service user researchers. We found trust was essential linked to relationship building that underpinned all our work and needed continual attention as the team was reshaped by staff departures and new researchers arriving. LEAP members and researchers needed to feel part of a safe and comfortable group before revealing aspects of their past, and current, lives. Projects must take time to build and maintain trusting relationships which focus both on the interpersonal and the practical.

The space that service user researchers worked in was challenging in PARTNERS2, impacted by research hierarchies that do not encourage power-sharing [31]. LEAP members valued those in the research team who drew upon lived experience, describing this as a bridge to their own role as advisors and they identified both small and major contributions which flowed from researchers’ dual expertise as ‘insiders’ to both being a service user and an academic. However, service user researchers had varied experiences, some concluding the role did not work, others acknowledging the compromises they had to make viewing decision making from dual perspectives. Service user researchers were collaborators with co-applicants and other research assistants. The term ‘involvement’ is insufficient to describe the work they undertook as research leaders. The role of service user researchers is closer to a co-production ethos, and a LEAP less so. This mix of roles allowed for greater variety and impact of lived experience input, however role descriptions and tasks should be accounted for and communicated well to teams to improve recognition of impact.

In PARTNERS2, we were unprepared for the ‘them and us’ culture that we unintentionally created in work that was grounded in principles of equality. This was most obvious in the paid research roles. It became apparent that several of our ‘academic researchers’ also had lived experience of mental health needs. We did not openly discuss with researchers their own lived experience and how they wanted to use it sufficiently in research tasks. As a result, valuable expertise remained hidden.

### Our reflections: PPI and co-production

From our work, we have seen how a co-production approach supports involvement work, but as others have found [22] this way of working involves significant challenges for research teams as they attempt to address power imbalances inherent within the research process and between individuals [23]. This complements research that conceptualises ‘involvement spaces’ as working ‘in-between’, within liminal spaces for the generation of public perspectives compared to professional/clinical/academic viewpoints [32]. Does partially employing co-production count as a failure? We do not believe so, considering the elasticity of the term co-production [33]. Being guided by PPI and co-production values and acting as both a collaborator and disrupter within a project, does not mean all aspects of an involvement research model are fully achieved. However, we found important parts of this way of working were transparency, clear communication, and a commitment to reflection and learning.

PPI and co-production approaches can both struggle with representation and representativeness [34]. Our work was not overly concerned with representation, instead seeking diversity of perspectives within LEAPs and across the service user researcher posts. Identities are by nature intersectional, complex and combine visible and invisible aspects. People joining PARTNERS2 brought multiple identities and experiences that were much richer than those tied solely to mental health. Mental health research studies could benefit from a greater focus on intersectional experiential expertise across the entire team. Our current thinking is that co-production, done well, is more inclusive, less tokenistic and offers more opportunities for meaningful use of expertise from experience than an approach based only on PPI. However, fundamentally research teams need be transparent about their approach, with research leaders open about their plans – be those PPI, coproduction or both - including limitations.

### Limitations

Apart from the PPI lead, this paper did not include reflections from PARTNERS2 co-applicant staff or other senior team members. We focused on working with those who were most engaged with PPI and co-production. Those not involved may have very different perspectives. Although we have aimed to act with reflexivity, we acknowledge our own interests in demonstrating successful involvement work within PARTNERS2.

## Conclusions

Experiential expertise is a part of PPI and co-production in research but few examples documenting collaborative research methods in practice exist. The learning in this paper is contextual within one large-scale research programme, but applicable to other studies. We demonstrate that PPI and co-production can be complicated and ‘messy’, but also show successes are achievable. For success, we found there needs to be a greater emphasis on the importance of emotional work, creating safe spaces to co-produce, transparency in decision making and reflection on the difficulties of using personal identities in research work including for service user researchers. These elements are more important than existing guidelines suggest. Implementation of actions to support emotional work, will require changes within individual teams as well as institutions. Research funders should also drive change more consciously, including balancing the entrenched prioritisation of clinical and research expertise over experiential expertise. Introducing reflective practice in teams may be helpful in identifying further improvements to support more inclusive research practice.

## Supplementary information

**Additional file 1.** **Appendix 1:** Reflective writing accounts about working on PARTNERS2.

## Data Availability

The material that forms the basis of this paper are provided in Appendix 1 in full.

## Abbreviations

LEAP: Lived Experience Advisory Group
NIHR: National Institute of Health Research
NSUN: National Survivor User Network
PPI: Patient and Public Involvement
SURA: Service User Research Assistant
