# Supplementary material for: Exploring patient and public involvement (PPI) and co-production approaches in mental health research: learning from the PARTNERS2 research programme
Source: Res Involv Engagem. 2020 Sep 21;6:56. doi: 10.1186/s40900-020-00224-3 (PMC7507647; doi:10.1186/s40900-020-00224-3)
Supplement: Supplementary file 1 — Additional file 1. Appendix 1: Reflective writing accounts about working on PARTNERS2. [file 40900_2020_224_MOESM1_ESM.docx]

***Appendix 1: Reflective writing accounts about working on PARTNERS2***

**Authors have coded their own accounts. See page 13 for coding summary.**

**Columns 1-3 show how each was coded by its author, or author group, against three sets of published NIHR INVOLVE standards or guidelines for PPI (codes 1-6) and coproduction two sets of codes 1-5 and 1-8. Column 4, titled themes, shows how each account links to our writing as described in the main results section (Themes A, B, C, D, E) provided for the accounts and tips provided by each author or author group.**

| ***Reflective Account A – from PPI Lead*** | ***PPI*** | ***CPP*** | ***CPKF*** | ***Themes*** |  |
| --- | --- | --- | --- | --- | --- |
| ***Integrating expertise from experience in a multi-site mental health research study – from theory to practice.*** | | | | |  |
| *A well-resourced plan was developed to ensure service users and carers were meaningfully involved in PARTNERS2, at every level. This was relatively straightforward, but delivery was not.  Why?*  *From our perspective it’s partly because of the complexity of working across three research sites, primary and secondary NHS systems, and the volume of people to keep ‘involved’ in ways that respect and value relevant expertise, knowledge and practical issues such as capacity.*  *But another challenge for collaboration in a research context are inherent, powerful and culturally embedded research hierarchies.*  *We’ve had some successes: bringing senior academics and LEAP members together within a ‘Taster’ session at the start ; altering seating arrangements in team meetings to mix people up; exercises to equalise power in meetings, sharing personal interests and skills; collecting feedback to revise processes; having PPI specified on every management agenda; acknowledging hidden personal experience of mental health problems within the team; focusing on clinical, research and experiential expertise as three equal parts of the PARTNERS2 resource bank.*  *Individual ‘breakthroughs’ in reciprocity and relationship building can feel short-lived when thinking about the entire programme including a randomised control trial. We have no shortage of ideas or goodwill but changing research cultures fundamentally remains a huge challenge.* | *2*  *6* | *1*  *2*  *3*  *4*  *5* | *4* | *Topic*  *C*  *D*  *Tip*  *C* |  |
| ***Tip: Keep developing new ways to develop PPI and service user research roles in practice in order to coproduce knowledge. Work together to accommodate the needs and sensitivities of everyone working in the research ecosystem.*** | | | | |  |
| ***Account B – From a LEAP (Lived Experience Advisory Panel) member*** | ***PPI*** | ***CPP*** | ***CPKF*** | ***Themes*** |  |
| ***Bridging the gap: the important role of service user researchers*** | | | | |  |
| *I am not an academic. I am a mother wading my way through the mental health system, so it’s an emotional journey too. Collecting data and writing papers aren’t my skills nor do I want them to be. I have more impact talking with people, sharing my knowledge and experience.*  *However, I feel it’s absolutely crucial we have service user researchers on board directly. Roles that form a bridge between service users/carers and academics. They understand where we are coming from. They explain things differently (more simply) offering examples from their direct experience. It could be said they wear two hats, bringing our motivational differences together.*  *The conduct of meetings are formal in that we have an agenda, yet informal, so I don’t feel pressured to speak a certain (academic) language. We have created a safe and comfortable space for discussions.*  *Maybe our LEAP members could start chairing meetings. This would help address the inherent power differentials between academics and LEAP members. We know teams try to re-balance things through paying us for our time, inviting us to author papers but still more could be done, and I think the service user researcher role reduces the gap between us and them in important ways.* | *2*  *3* | *1*  *2*  *3*  *4* | *2*  *4*  *5* | *Topic*  *A*  *B*  *C*  *D*  *Tip*  *C* |  |
| ***Tip: Develop the roles for service user and carer advisors and service user researchers further by continuing to provide opportunities for coproduction.*** | | | | |  |
|  | | | | |  |
| ***Account C – From a LEAP member*** | ***PPI*** | ***CPP*** | ***CPKF*** | ***Themes*** |  |
| ***Accessibility and culture/ethos of LEAP available support - personalised and just in time.*** | | | | |  |
| *I was approached by another carer involved in PARTNERS2. She gave me the encouragement to become part of a LEAP to work towards collaborative mental health care in primary care and CMHTs.*  *Anxiety disorder and depression have previously prevented me commuting to work and gaining academic qualifications in a research profession. PARTNERS2 has removed these barriers for me to feel empowered and ask for assistance with travel practicalities and gain timely reimbursement for expenses.*  *As patient and carer involvement in research transforms and becomes fundamental to design and delivery of research, it is essential that there is improvement around accessibility, with consideration and flexibility to how individuals can be supported to attend and participate safely and confidently.*    *Involvement has led onto other work within PARTNERS2. It has enabled me to add value to my difficult health experiences and positively redefine 'mental illness'', overcoming the detachment from a working society.*  *It is important to collaborate with researchers that have health challenges enabling rapport building and create a unique relationship, giving greater opportunity for accessibility into patient and public involvement.* | *1*  *2* | *2*  *2*  *5* | *2*  *4*  *5*  *6*  *7* | *Topic*  *B*  *C*  *Tip*  *C* |  |
| ***Tip: Build on a person-centred approach when encouraging and actively listening to service users and carers involved in research.*** | | | | |  |
|  | | | | |  |
| ***Account D – From a LEAP member*** | ***PPI*** | ***CPP*** | ***CPKF*** | ***Themes*** |  |
| ***Use of personal experience in PARTNERS2: when to disclose and when not.*** | | | | |  |
| *I don’t think of myself in a carer role; but I do care deeply for the members of my family who have been affected by mental illness. When a friend passed on the opportunity to be a member of a research advisory group, I welcomed the opportunity to do something that was not about “being in the illness”.*  *I have gained much from being involved in this project and learnt through some challenges. I was there because of my experience; I didn’t see it as being about sharing personal information.*  *I remember being frustrated with one member sharing details about their family member. I did not want to hear about their problems. I felt very judgemental and resentful that they were taking up time sharing; I did not think it was what we were there for.*  *What could I do with these feelings? I realised that I had set the rules about ‘no sharing’. I had to respect that the other member wanted to share. We were there because of our personal experience, relevant for professional research.*  *I learnt to develop respect for the differences that existed and for some that might mean more disclosure than for others.* | *1*  *2* | *2*  *3*  *5* | *2*  *4*  *5*  *6*  *7* | *Topic*  *A*  *C*  *Tip*  *A* |  |
| ***Tip: Co-produce guidelines on disclosure and how to use lived experience in a project, for all those who bring experiential expertise to a research programme.*** | | | | |  |
|  | | | | |  |
| ***Account E – From a LEAP member*** | ***PPI*** | ***CPP*** | ***CPKF*** | ***Themes*** |  |
| ***The value of multiple identities and creating safe spaces for involvement*** | | | | |  |
| *I joined a PARTNERS2 LEAP as someone who has used mental health services, but I also brought other identities, some more visible than others: carer for my Mum, ethnic minority background, gay man, Muslim.*  *In LEAP meetings my mental health identity takes over, but people forget that mental ill health and wellbeing is affected by all of our identities. So the research will benefit from including all those perspectives but only if I feel safe and can choose whether and how to reveal them.*  *I have moved LEAPs during the project so have experienced membership of two separate groups. The first LEAP felt like a family and was based in a community venue, almost like walking into a neighbour’s house. It felt safe, friendly and open, people brought personal issues to the discussion, strong social connections were established, and conversations were therapeutic. I feel my sexuality was appreciated, acknowledged, and celebrated. The second LEAP feels much more formal. Meetings are held on secure university premises. We work together as colleagues, rather than family.*  *Both LEAPs have enabled me to bring personal experience to the research tasks, but in different ways. My skills, expertise and multiple identifies add value, but these could be overlooked if not nurtured and valued by researchers.* | *1*  *2*  *3*  *4*  *5* | *2*  *3*  *4*  *5* | *2*  *4*  *5*  *6* | *Topic*  *B*  *A*  *Tip*  *A* |  |
| ***Tip: Do not under-estimate the value of social connection and deeper meaningful conversations: these will strengthen PPI by developing trust and confidence across a whole team.*** | | | | |  |
|  | | | | |  |
| ***Account F – From a LEAP member and SURA (Service User Research Assistant)*** | ***PPI*** | ***CPP*** | ***CPKF*** | ***Themes*** |  |
| ***A Critical Friend and Colleague: from LEAP member to Service User Researcher*** | | | | |  |
| *I have worked on PARTNERS2 as both a LEAP member and Service User Researcher, giving me two distinct experiences of how decisions are reached.*  *Firstly, a LEAP member, I was able to have a critical distance from the project, attending meetings quarterly, enjoying a ‘snapshot’ and making useful suggestions that might otherwise have been passed over by the research team. The key word as a LEAP member is ‘Advisory.’ I was consulted on a regular basis and my input, along with that of others, was valued and respected, even if in the end it was not adopted. I was aware of a research hierarchy in PARTNERS2 but still felt part of the process.*  *Later, as a Service User Researcher, my role has varied. I draw on my personal experiences to contribute to many aspects of the study, as well as carrying out the tasks of an academic researcher.*  *I am much closer to day-to-day decision-making. In theory, I have more ‘power’ to shape and direct the project.*  *However, the practicalities of my role have made me far more aware of the need for compromise and pragmatism. We seek collaborative and transparent decision-making, but can feel disconnected and disempowered by decisions taken.* | *2*  *4*  *6* | *2*  *3*  *4*  *5* | *1*  *2*  *4*  *5*  *6* | *Topic*  *D*  *C*  *Tip*  *C* |  |
| ***Tip: Encourage more people to move from advisory to research roles and vice versa as both play an important part in complex mental health research trials.*** | | | | |  |
|  | | | | |  |
| ***Account G – From PPI coordinator and Peer Researcher*** | ***PPI*** | ***CPP*** | ***CPKF*** | ***Themes*** |  |
| ***Using my personal and professional expertise: advocate, interpreter, facilitator?*** | | | | |  |
| *Within this job I’ve worn two hats – coordinating PPI and LEAP work, and contributing my bipolar experience as peer researcher. Both have been challenging in different ways, at different points, and immensely satisfying at other points. For me, a roller-coaster ride with handrails that, at first, were not yet screwed in properly. I fell 3 times, went off to lick my wounds in private.*  *But, from every bump and crash, we learned how to find our balance and not risk tipping the whole thing over. Learning to be flexible, but mindfully.*  *My first work meeting was with the Workstream 2 researchers developing Core Outcome Sets (COS) for Bipolar and Schizophrenia. This struck me as a daunting but vitally important task.*  *However, we were starting from different perspectives and experiences - of methodologies, psychiatry and activism. We began by discussing language and approach to “psychiatric patients” - how might they differ from other patient groups? I advocated for broader conceptualisation - from “illness” towards “wellbeing”. Basic concepts had to be accessible to focus group recruits. “What outcomes are important?” became “What would you hope your mental health support might enable you to do, or to achieve?”*  *Following the COMET protocols over three years, we arrived at a COS for Bipolar, and await publication.* | *1*  *2*  *3*  *4*  *5* | *1*  *2*  *3*  *4*  *5* | *1*  *3*  *5*  *6*  *7*  *8* | *Topic*  *A*  *C*  *Tip*  *C* |  |
| ***Tip: Accept and value all of the expertise available in a research team. We can all learn from listening closely to each other’s voices*** | | | | |  |
|  | | | | |  |
| ***Account H – From two LEAP members*** | ***PPI*** | ***CPP*** | ***CPKF*** | ***Themes*** |  |
| ***The value of reciprocity within a research study*** | | | | |  |
| *Involvement work in research benefits the ‘public’, e.g. PARTNERS2 service users or carers with personal experience of schizophrenia or bipolar illness, and academic researchers. The interaction helps link different experts together to transform mental health practice.*  *As LEAP members we were viewed as having skills and a unique perspective. Engaging with each other, researchers, and administrative staff, produced new insights, trust-building and created a positive working environment.*  *We felt valued by payment for our time. We also learnt from each other and acquired skills to use in other roles, including trustee of a local drop-in centre. We took part in study milestones such as drafting important outcomes for bipolar and schizophrenia by sharing our knowledge and experiences. We helped write a guide for carers about the new service.*  *It was empowering but we could bridge the gap even further between academics and people with personal experience of the topic being researched. We saw few senior staff at the LEAP meetings, which was unfortunate as we could have learned a lot from each other.*  *This work is innovative and challenging. Shaping the project was our goal, knowing each of us had something important to offer. Our LEAP feels like a team.* | *1*  *2*  *3*  *4*  *6* | *1*  *2*  *3*  *4*  *5* | *1*  *2*  *3*  *4*  *5*  *8* | *Topic*  *C*  *B*  *D*  *Tip*  *C* |  |
| ***Tip: Actively develop reciprocal learning environments, which allow service users and carers alongside researchers to share knowledge, develop new skills and research expertise.*** | | | | |  |
|  | | | | |  |
| ***Account I – From Research Fellow*** | ***PPI*** | ***CPP*** | ***CPKF*** | ***Themes*** |  |
| ***The practical impact of a LEAP involvement: supporting a researcher’s understanding of health service endings*** | | | | |  |
| *Over the four years of my role in PARTNERS, I saw the LEAPs develop from individuals to an integrated, effective community. My role involved the development of a practitioner manual. Consulting with LEAP members was part of this process. Although these sessions weren’t always easy - I felt judged as an academic for not being ‘one of them’ - their input was valuable.*  *A LEAP meeting in Exeter developed my empathy about the experience of being transferred to a new health practitioner. While I had understood the issues from reading literature, LEAP members’ stories about losing support that they depended on, and distress over losing relationships with trusted practitioners, brought the issue to sharp focus.*  *Later, I asked the Birmingham LEAP for feedback about practitioner guidelines for transfer in the manual. They emphasised the need for substantial changes, notably working in partnership to plan the final review, and providing sensitising statements about transfers.*  *I found the comments difficult to hear – I think because I had worked hard on the resources following the empathetic stance I developed in Devon. It was humbling to see the benefit of the changes I had been blind to before the LEAP suggested them.* | *2*  *5* | *1*  *3*  *5* | *3*  *4*  *8* | *Topic*  *C*  *A*  *B*  *Tip*  *C* |  |
| ***Tip: Respect LEAP members’ knowledge and experience – this was fundamental to improving PARTNERS2 in meaningful ways*** | | | | |  |
|  | | | | |  |
| ***Account J – From two LEAP members*** | ***PPI*** | ***CPP*** | ***CPKF*** | ***Themes*** |  |
| ***Developing confidence to provide meaningful involvement.*** | | | | |  |
| *As members of Devon LEAP we helped choose the primary and secondary trial outcome measures. This is a good example of collaboration, supported by researchers at every stage.*  *The process started in 2015 with LEAP conversations in all three groups, and finished with an interview trialling them individually with LEAP volunteers in 2017. As LEAP members we didn’t make the final decision but our input shaped the selection process.*    *Initially xxx was unfamiliar with the concept of ‘Outcome Measures’ and felt 'overwhelmed'. Beverly had previous experience of outcome measures in a different context. During the process, our knowledge and sense of value within the project shifted.*  *"My confidence increased and I felt an active part of the project".*  *"I was really able to contribute, as I could empathise with PARTNERS2 participants and consider what might be best for them. I found the whole experience interesting, and very informative".*  *We both agree there were definite benefits from the service user participation in shaping the project delivery, in a way that acknowledges the complexity of the issues involved.*  *Our contribution as LEAP members in deciding the outcomes and conclusions of this phase of the project was definitely valid and valued.* | *1*  *6* | *2*  *3* | *2*  *3*  *5*  *8* | *Topic*  *A*  *C*  *B*  *Tip*  *C* |  |
| ***Tip: Provide initial training and preparation on the research process for LEAP members. Value the contribution of service users and all the progression made*** | | | | |  |
|  | | | | |  |
| ***Account K – From a LEAP member*** | ***PPI*** | ***CPP*** | ***CPKF*** | ***Themes*** |  |
| ***Messiness of PPI in Partners2.*** | | | | |  |
| *I guess any project like PARTNERS2 is very complex and messy, and it certainly has been. From sitting in a hot stuffy room at Birmingham University taking part in a ‘taster’ information session, to the many hours spent trying to decide on outcomes for the Bipolar Core Outcomes Set, a lot of work has gone on.*    *At times trying to keep on track of which work stream we were commenting upon and what all the different terms meant was complex and difficult, at times I felt hugely out of my depth. But I have also contributed a lot, writing notes after each LEAP meeting. I received lots of positive feedback after chairing our last session.*  *Being a member of the LEAP involves compromises. I have felt conflicted in the decisions I was asked to make. The day that felt the hardest to me was one of the joint meetings – LEAP members and academics. We were trying to decide on which outcome questionnaires participants would be completing. I felt very conflicted.*  *One of the favoured questionnaire asked about work and claiming benefits. I could see from a researcher point of view why this choice made sense. But I felt I was betraying service users because recording outcomes about benefits and work related issues can cause, and raise fears that people might lose benefits. This is just one example of the messiness of working with clinicians, academic and patients and carers. This is just one example of the messiness of working with clinicians, academic and patients and carers.* | *1*  *2*  *3*  *6* | *1*  *2*  *3* | *3*  *6* | *Topic*  *C*  *A*  *Tip*  *A* |  |
| ***Tip: Before involving service users and carers in complex trials, consider carefully what training and support can be provided to help with pragmatic decision-making and deal with any conflicted feelings.*** | | | | |  |
|  | | | | |  |
| ***Account L – From two LEAP members and a Research Assistant*** | | ***PPI*** | ***CPP*** | ***CPKF*** | ***Themes*** |
| ***How are decisions made in PARTNERS2?*** | | | | | |
| *LEAPs work alongside researchers on PARTNERS2, informing the study through the input of ‘expertise from experience’. LEAP members were included at all stages of the project, but as always the research process gave members the impression of being listened to but not having the final say. This was partly due to the logistics of quarterly meetings, but also a result of academic hierarchy.*  *One example of collaboration between the researchers and the LEAPs was throughout the design of the PARTNERS2 participant website. This was designed by the research team. All three LEAPs provided feedback and had a substantial impact on all aspects, including writing scripts for videos and recording them.*  *This reinforced the importance of expert stakeholder involvement in research, as the input of service users and carers in the website helped validate the research and ensure it was suitable for potential participants.*  *However, shared decision-making (as traditionally described by health services) was limited to the initial stages, with research leads making all final approvals.*  *Where possible, projects should strive towards mutual agreement between academic experts and those with ‘expertise from experience’. This reduces the perception of the superiority of academic and clinical knowledge over experiential knowledge.* | | *2*  *4*  *6* | *1*  *2*  *3*  *5* | *1*  *2*  *3*  *4* | *Topic*  *D*  *C*  *Tip*  *D* |
| ***Tip: Be transparent within any research as to which model of decision-making is being used and why decisions are made, in order to help meet the principles of coproduction*** | | | | | |
|  | | | | | |
| ***Account M – from three Research Assistants*** | | ***PPI*** | ***CPP*** | ***CPKF*** | ***Themes*** |
| ***Moving beyond the title of ‘service user researcher’: a step towards co-production.*** | | | | | |
| *There was a sense that the title of ‘service user researcher’ was too narrow. It could be detrimental to a researcher’s career, and lead to ignoring other qualifications and experience. It did not work because we found that many people in our team across all three sites had lived experience.*  *We have dropped the title in the South West. All new appointments are ‘research assistants’. We share our workload equally, including lived experience where the researcher wants to.*  *This feels like a move towards coproduction across the sites, but especially in our site. It includes all perspectives and skills, and experience that remains ‘undisclosed’, like personal difficulties with mental health that a team member might choose not to declare publicly.*  *This is an example of respecting and valuing the knowledge of all those working together on the research, including those drawing on experience of poor mental health.* | | *1*  *2*  *3* | *2*  *3* | *2*  *6*  *7* | *Topic*  *C*  *D*  *Tip*  *C* |
| ***Tip: Recognise that most people will have been affected by poor mental health - we should guard against creating an ‘us and them’ approach in collaborative teams.*** | | | | | |
|  | | | | | |
| ***Account N – From a SURA*** | | ***PPI*** | ***CPP*** | ***CPKF*** | ***Themes*** |
| ***Inside-Outside or somewhere in-between*** | | | | | |
| *Whilst not being something I’ve ever denied, the role and identity of being a ‘service user researcher’ on PARTNERS2, has at times created an internal tension for me, particularly with the transition of the project to a site where I’d previously accessed secondary care services.*  *Once I would have been the one waiting in the reception area of the CMHT, I have felt at times that I am now ‘on the other side’ with my own swipe card to access the inner sanctum of the building.*  *Being a ‘service user researcher’ has often seemed to work in many different and contrary ways within PARTNERS2. Sometimes as an asset, one example being a resource to draw upon in conversations with potential participants, including participating GP practices.*  *At other times there has been a sense of appearing somehow of less ‘status’ within the academic hierarchy.*  *These tensions perhaps begin to highlight the inherent contradictions that both explicit and implicit personal experience of mental health issues, can evoke across different contexts.* | | *2*  *3*  *5* | *2*  *3*  *5* | *1*  *2*  *3*  *4*  *5*  *6*  *7*  *8* | *Topic*  *C*  *A*  *Tip*  *A* |
| ***Tip: Consider carefully the potential impact of working in a research environment that may have significant personal or emotional resonance, and negotiate a process to manage this.*** | | | | | |
|  | | | | |  |
| ***Account O – From a Research Assistant*** | | ***PPI*** | ***CPP*** | ***CPKF*** | ***Themes*** |
| ***Being Inside Out – the researcher in PARTNERS2*** | | | | | |
| *My job title was as a research assistant on PARTNERS2. Being labelled as a non-service user researcher has meant pushing back against the power of being as a ‘normal’ researcher, and not wanting to impose that traditional research identity upon people with lived experience in our local LEAP. That went against the ethos of the project and felt uncomfortable personally.*  *However, being labelled as a “normal” researcher negated my own insights from actually having mental health experiences - simply because it wasn’t in my job title or expected of me.*  *This made me feel uneasy in the sense of being labelled as an outsider in our LEAP, by people in the group stereotypically labelled as outsiders themselves, whilst also actually having my own lived experience.*  *This has meant reflecting on what a LEAP member’s view of me could be, and positioning myself in the role as a “normal” researcher. None of this was ever discussed openly.*  *I assumed a role of academic researcher and felt both connection and disconnection to other people around me. There was so much discussion and work around PPI in the project that this position of being “Inside Out” felt very strange.* | | *1*  *2* | *2*  *3*  *4*  *5* | *1*  *2*  *5*  *6* | *Topic*  *B*  *C*  *D*  *A*  *Tip*  *C* |
| ***Tip: Explicitly state that many people have a mental health diagnosis at the start of meetings, to demonstrate that there is no ‘us and them’ distinction. Discuss and manage expectations of roles with other team members to use all expertise fruitfully.*** | | | | | |

***Key to Coding of the 4 columns***

***Patient and public involvement standards (PPI)***

1. *Inclusive opportunities - clear, meaningful and accessible opportunities for involvement, for a wide range of people across all research.*
2. *Working together - create and sustain respectful relationships, policies, practices and environments for effective working in research.*
3. *Support and learning - ensure public involvement is undertaken with confidence and competence by everyone.*
4. *Communications - provide clear and regular communications as part of all involvement plans and activities.*
5. *Impact - assess report and act on the impact of involving the public in research.*
6. *Governance - ensure the community of interest voices are heard, valued, and included in decision-making.*

***Coproduction – principles (CPP)***

1. *The sharing of power, demonstrated through decision-making*
2. *Inclusivity of approach, drawing on the perspectives of everyone who can make a contribution*
3. *Respecting and valuing the knowledge of all those working on the research project*
4. *Reciprocity, shared benefits of working together are realised by all*
5. *Building and maintaining relationships is central to co-production*

***Coproduction – key features (CPF)***

1. *Establishing ground rules*
2. *Ongoing Dialogue*
3. *Joint ownership of key decisions*
4. *A commitment to relationship building*
5. *Opportunities for personal growth and development*
6. *Flexibility*
7. *Continuous reflection*
8. *Valuing and evaluating the impact of co-producing research*

***Our paper themes – coding emerging from reading the reflective writing accounts***

1. Recognising the importance of emotional work
2. Developing safe spaces to create and share knowledge
3. Understanding challenges of using personal identity in research work
4. Working to share power within a research hierarchy
